# Risk factors for intrahepatic cholangiocarcinoma: a possible role of hepatitis B virus

**DOI:** 10.1111/j.1365-2893.2009.01243.x

**Published:** 2010-10

**Authors:** M Tanaka, H Tanaka, H Tsukuma, A Ioka, A Oshima, T Nakahara

**Affiliations:** 1 Department of Cancer Control and Statistics, Osaka Medical Center for Cancer and Cardiovascular DiseasesOsaka, Japan; 2 Aichi Cancer Center Research InstituteNagoya, Japan; 3 Cancer Information Services, Osaka Medical Center for Cancer and Cardiovascular DiseasesOsaka, Japan; 4 Graduate School of Medicine, Kyoto UniversityKyoto, Japan

**Keywords:** aetiology, cholangiocarcinoma, cohort studies, hepatitis, inflammation, liver

## Abstract

There are several established risk factors for intrahepatic cholangiocarcinoma (ICC), namely primary sclerosing cholangitis, fibropolycystic liver disease, parasitic infection, intrahepatic biliary stones and chemical carcinogen exposure. However, the majority of patients with ICC do not have any of these risk factors. Therefore, identification of other risk factors is warranted for the prevention and early detection of ICC. We evaluated the risk factors for ICC in a large-scale cohort study in the province of Osaka, Japan. This retrospective cohort study included 154,814 apparently healthy individual blood donors, aged 40–64 years at the time of blood donation in the period 1991–1993. The average observation period was 7.6 years, resulting in 1.25 million person-years of observation. Incident ICC cases were identified by linking the blood-donor database to the records in the population-based cancer registry for the province. There were 11 incident ICC cases during follow-up, with an incidence rate of 0.88 per 100 000 person-years. Compared with subjects aged 40–49 years, the subjects aged 50–54 years and 55–59 years had a significantly higher risk for ICC (hazard ratio [HR] = 5.90; 95%CI:1.08–32.31 and 11.07; 95%CI:1.98–61.79, respectively). Compared with those with ALT level of 19 Karmen Units (KU) or less, subjects with ALT level of 40 KU or higher had a significantly higher risk for ICC (HR: 8.30; 95%CI:1.47–46.83). Compared with those who tested negative for both HBsAg and anti-HCV, those who tested HBsAg-positive had a significantly higher risk for ICC (HR: 8.56; 95%CI: 1.33–55.20). Our results suggest that HBV infection and liver inflammation are independently associated with ICC development. These findings need to be verified by further large cohort studies.

## Introduction

Intrahepatic cholangiocarcinoma (ICC) is the second most common primary liver cancer, accounting for approximately 10–20% of liver cancers [1]. Worldwide, it is estimated to account for 3% of all gastrointestinal cancers [2]. Advanced ICC has a very poor prognosis with a median survival of less than 24 months [3]. There is extensive variation among the incidence rates of ICC in different parts of the world, and the incidence is reported to be higher in East Asia [1]. The reported incidence of ICC in several developed countries has been increasing in recent years [1,4,5].

There are several established risk factors for ICC [1,4], namely primary sclerosing cholangitis, fibropolycystic liver disease, parasitic infection, intrahepatic biliary stones and chemical carcinogen exposure, but the majority of patients with ICC do not have any of these risk factors. Therefore, identification of other risk factors is warranted for the prevention and early detection of ICC. In addition to the established risk factors mentioned earlier, some other potential risk factors for ICC have been suggested, such as infection with hepatitis B virus (HBV) [6–9], hepatitis C virus (HCV) [9–14] or liver cirrhosis [7,11,12]. Almost all of the studies that suggested these potential risk factors, however, were case–control studies. Also, half of the reported studies were conducted in the USA or Italy, and their results may not be applicable for populations in other countries or areas, partly because the relative importance of risk factors may vary by country or by area. To our knowledge, no cohort study has been reported from a non-Western country on the risk factors for ICC. In this background, we conducted a retrospective cohort study in Japan, using a large group of apparently healthy blood donors, to assess the incidence and risk factors for ICC.

## Materials and methods

### Subjects

The subjects were those who were involved in our previous study on the risk of developing hepatocellular carcinoma, which was reported elsewhere [15]. They were selected from voluntary blood donors who gave 1 235 926 allogeneic blood donations at the Osaka Red Cross Blood Center between 1991 and 1993. The blood centre is in charge of managing volunteer blood donations in the province of Osaka, which had a population of approximately 8.6 million during this period. Conditions required for potential blood donors in Japan during the study period were described elsewhere [16]. In brief, they had to be aged between 16 and 64 years and have a haemoglobin level of 12 g/dL or higher. Potential blood donors were preliminarily screened by a self-administered questionnaire with items regarding past and present illnesses, history of blood transfusion, illegal drug use and high-risk sexual behaviours. Major exclusion criteria in the preliminary screening included: (i) known or potential infection with HBV, HCV, human immunodeficiency virus (HIV) or human T-cell lymphotropic virus type 1 (HTLV-1); (ii) presence or history of chronic liver disorders as well as malignant, allergic or autoimmune disorders; (iii) history of illegal drug use or high-risk sexual behaviour. Those who did not meet any of the exclusion criteria in the preliminary screening donated blood without monetary incentive. The donated blood of these donors was tested for the earlier-described viruses, and, if tested positive, the blood was discarded, but the information on the positive-tested donors was kept in the blood-donor database as was the information on the negative-tested donors. Using this database containing information on all of the donors and the results of the serologic screening test, we identified 667 461 individual donors by donor birthdate, sex, first name, family name and ABO blood type. From these individuals, we selected residents in Osaka province, aged 40 years or older at the time of blood donation, who were negative for antibodies to HIV and HTLV-1. Those who were 39 years or younger were excluded because the incidence of ICC in this age-group in the Japanese population was negligible. Those infected with HCV or HBV were first-time blood donors who claimed to be asymptomatic at the time of blood donation. Those who were infected with both HBV and HCV (25 subjects) were also excluded. As a result, we identified 154 814 persons to be included in this cohort study.

### Blood screening tests

The serum alanine aminotransferase (ALT) level expressed in Karmen Units (KU) and serum total cholesterol level (mg/dL) were obtained from the database. The ALT value in KU tested by the blood centre can be translated into a value in International Units by multiplying with 1.5 [17]. An individual was defined as having HCV infection if the titre of anti-HCV in a second-generation passive hemagglutination assay (PHA) (Dainabot Co., Ltd, Tokyo, Japan) was 2^12^ or higher, because the positive predictive value for being HCV-RNA-positive using this cut-off point in Japanese blood donors was guaranteed [18]. An individual was considered to have HBV infection if he/she was positive for HBsAg in a reverse passive hemagglutination assay (Japan Red Cross, Tokyo, Japan).

### Follow-up

The subjects were followed-up by record linkage between the blood-donor database and the database of the Osaka Cancer Registry (OCR) [19]. The records in the two databases were linked by the parameters of sex, date of birth, address and the first character of the family name in Chinese letters. The OCR is a population-based cancer registry that covers all of the population in Osaka province. The OCR registers all incident cancer cases using reports from health care facilities in the province as well as death certificate information provided by the Osaka Provincial Government [19]. In the OCR database, ICC cases were identified by the ICD-10 code (C22.1). The diagnosis of ICC was based on histological examination and/or combined clinical, radiological (echography, computed tomography and endoscopic retrograde cholangio-pancreatography) and laboratory findings. Subjects who remained unaffected by ICC were censored at the last date of follow-up, 31 December 2000, as were the subjects in the previous study [15]. The study protocol was approved by both the Ethical Committee of Osaka Medical Center for Cancer and Cardiovascular Diseases, and the Ethical Committee of the Osaka Red Cross Blood Center.

### Statistical analyses

The number of person-years of observation of the subjects was determined, and the incidence rate of ICC per 100 000 person-years was calculated by the strata of age-group, sex, ALT level, cholesterol level and HBV/HCV infection status. Ninety-five per cent confidence interval (95%CI) for the rate was calculated using Byar’s approximation of the exact Poisson test. Independent factors associated with the development of ICC were analysed by Cox proportional hazards model, and hazard ratios were calculated with 95%CI. In the model, age-group, sex, ALT level, cholesterol level, and HBV/HCV infection were included as independent variables. Data analyses were performed with the SAS/PC statistical package (SAS Institute, Cary, NC, USA).

## Results

The baseline characteristics of the 154 814 study subjects and incidence rates of ICC stratified by different characteristics are shown in Table 1. The proportions of those with ALT level of 40 KU or higher, with positive HBsAg test, and with positive anti-HCV test were 2.2%, 1.6% and 1.2%, respectively. The average observation period was 7.6 years, resulting in 12.50 × 10^5^ person-years. There were 11 incident ICC cases, with an incidence rate of 0.88 per 100 000 person-years (95%CI: 0.44–1.58). By strata, the point-estimate incidence rate was higher among those aged 55–59 years, those with ALT level of 40 KU or higher, or those who tested positive for HBsAg or anti-HCV.

**Table 1:** Baseline characteristics of the study subjects of blood donors aged 40–64 years and incidence rates of intrahepatic cholangiocarcinoma.

	N	(%)	ICC incident cases	Person-years (×10^5^)	Incidence rate of ICC (per 10^5^ person-years)	95% Confidence interval* of ICC incidence rate (per 10^5^ person-years)
All cases	154 814	100.0	11	12.50	0.88	0.44–1.58
Age at blood donation (years)						
40–49	90 223	58.3	2	7.29	0.27	0.03–0.99
50–54	35 308	22.8	4	2.85	1.41	0.38–3.59
55–59	20 668	13.4	4	1.67	2.40	0.64–6.13
60–64	8615	5.6	1	0.69	1.44	0.02–8.06
Sex						
Male	84 205	54.4	7	6.81	1.03	0.41–2.12
Female	70 609	45.6	4	5.68	0.70	0.19–1.80
ALT (KU)						
19 or lower	127 757	82.5	7	10.32	0.68	0.27–1.40
20–39	23 666	15.3	2	1.91	1.05	0.12–3.78
40 or over	3391	2.2	2	0.27	7.36	0.83–26.74
Cholesterol (mg/dL)						
139 or lower	4533	2.9	0	0.37	0.00	0.00–12.60
140–199	82 575	53.3	8	6.67	1.20	0.52–2.36
200 or over	67 706	43.7	3	5.46	0.55	0.11–1.61
Hepatitis B/C virus infection						
HBsAg+	2519	1.6	2	0.22	9.08	1.02–32.82
anti-HCV+	1927	1.2	1	0.16	6.34	0.08–34.77
All negative	150 368	97.1	8	12.12	0.66	0.28–1.30

None of the subjects was positive for human immunodeficiency virus or human T-cell lymphotropic virus type 1. All negative: tested negative for both anti-HCV and HBsAg. ALT, alanine aminotransferase; HBsAg+, tested positive for Hepatitis B surface antigen and negative for Hepatitis C virus antibody; anti-HCV+, tested positive for anti Hepatitis C virus antibody and negative for Hepatitis B surface antigen. *95% confidence interval was calculated by Byar’s approximation of the exact Poisson test.

Factors associated with the development of ICC in blood donors are shown in Table 2. Compared with subjects aged 40–49 years, the subjects aged 50–54 years and 55–59 years had a significantly higher risk for ICC. Compared with those with ALT level of 19 KU or lower, subjects with ALT level of 40 KU or higher had a significantly higher risk for ICC. Compared with those who tested negative for both HBsAg and anti-HCV, those who tested positive for HBsAg had a significantly higher risk for ICC. The hazard ratio for anti-HCV positivity was 2.63, although it was not significant.

**Table 2:** Factors associated with the development of intrahepatic cholangiocarcinoma in blood donors according to Cox proportional hazard analysis

Variable	*n*	ICC	Hazard ratio	95%CI
Age at blood donation (years)				
40–49	90 223	2	1.00	
50–54	35 308	4	5.90	1.08–32.31
55–59	20 668	4	11.07	1.98–61.79
60–64	8615	1	6.61	0.59–74.59
Sex				
Male	84 205	7	1.00	
Female	70 609	4	0.79	0.22–2.82
ALT level (KU) at blood donation				
19 or lower	127 757	7	1.00	
20–39	23 666	2	1.47	0.29–7.36
40 or over	3391	2	8.30	1.47–46.83
Cholesterol level at blood donation*				
200 mg/dL or higher	67 706	3	1.00	
140–199	82 575	8	2.36	0.60–9.26
139 or lower	4533	0	–	–
Hepatitis B/C virus infection				
All negative	150 368	8	1.00	
HCV-Ab +	1927	1	2.63	0.25–27.73
HBs Ag+	2519	2	8.56	1.33–55.20

None of the subjects was positive for human immunodeficiency virus or human T-cell lymphotropic virus type 1. Age (4 categories), sex, serum ALT level at blood donation (3 categories), and serum cholesterol level at blood donation (3 categories) were included as independent variables in the Cox proportinal hazard analysis. ALT, alanine aminotransferase; Cl, confidence interval; HBsAg+, tested positive for Hepatitis B surface antigen and negative for Hepatitis C virus antibody; HCV-Ab+, tested positive for anti Hepatiis C virus antibody and negative for Hepatitis B surface antigen; ICC, intrahepatic cholangiocarcinoma; KU, Karmen Unit.

**Fig. 1:**
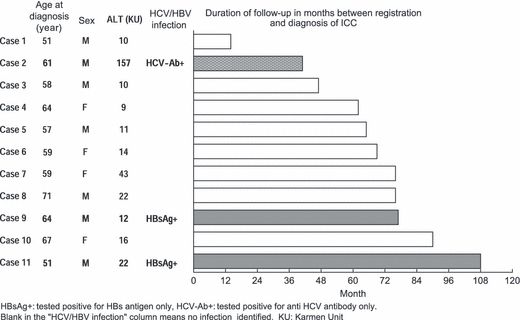
Characteristics of the 11 intrahepatic cholangiocarcinoma (ICC) cases identified among the cohort of blood donors during the follow-up period.

The characteristics of the 11 ICC cases identified during the follow-up period are summarized in Fig. 1. The observation period from the date of blood donation to the date of diagnosis of ICC ranged from 14 to 90 months among the eight cases not infected with HBV or HCV, while it was ‘41’, ‘77’ and ‘108’ months in the one HCV- and two HBV-infected cases.

## Discussion

To our knowledge, this is the first cohort study that investigated the risk factors for ICC in East Asia, where the incidence of primary liver cancer is high [20]. A literature search in Medline from January 1992 up to May 2009 revealed that all of the analytical studies on the risk factors for ICC in the past employed the case–control design, except for one study (Table 3). The only cohort study in the past was reported from the USA in early 2009 and focused on the risk of HCV infection for ICC and other hepatobiliary carcinomas [10]. The present study assessed whether HBV infection or liver inflammation as expressed by the ALT level was independently associated with ICC, which was not scrutinized in the cohort study from the USA.

**Table 3:** Results of analytical studies on the association of intrahepatic cholangiocarcinoma (ICC) and hepatits B and C virus

Authors and year	Study area	Type of study	No. of ICC case	RR (95% CI) for HBV infection*	RR (95% CI) for HCV infection*
Shin *et al.* 1996 [30]	Pusan, Korea	Case–control, hospital- based	41	OR = 1.3 (0.3–5.3)	OR = 3.9 (0.9–17.1)
Donato *et al.* 2001 [14]	Italy	Case–control, hospital- based	26	OR = 2.7 (0.4–18.5)	OR = 9.7 (1.6–58.9)
Yamamoto *et al.* 2004 [13]	Osaka, Japan	Case–control, hospital- based	50	OR = 1.8 (0.3–10.1)	OR = 16.8 (5.7–50.0)
Shaib *et al.* 2005 [12]	TX, USA	Case–control, Medicare- beneficiaries	625	OR = 0.8 (0.1–5.9)	OR = 6.1 (4.3–8.6)
Welzel *et al.* 2007 [11]	USA	Case–control, population-based	535	–	OR = 5.4 (2.9–10.2)
Shaib *et al.* 2007 [9]	TX, USA	Case–control, hospital-based	83	OR = 28.6 (3.9–1268.1)†	OR = 7.9 (1.3–84.5)
Lee *et al.* 2008 [7]	Korea	Case–control, hospital-based	622	OR = 2.3 (1.6–3.3)	OR = 1.0 (0.5–1.9)
Zhou *et al.* 2008 [8]	Shanghai, China	Case–control, hospital-based	312	OR = 8.8 (5.9–13.1)	OR = 0.9 (0.3–3.1)
Lee *et al.* 2009 [6]	Taiwan	Case–control, hospital-based	160	OR = 5.0 (2.8–9.0)	OR = 2.7 (1.2–6.3)
El-Serag *et al.* 2009 [10]	USA	Cohort, veterans population	37	–	HR = 2.6 (1.3–5.0)
Tanaka *et al.* (present)	Osaka, Japan	Cohort, blood donor population	11	HR = 8.6 (1.3–55.2)	HR = 2.6 (0.3–27.7)

Note: Hepatitis B virus infection status was identified by the presence of HBs antigen except the study by Shaib *et al.* 2007. (−): not asssessed as a covariate. Cl, confidence interval; HBV, hepatitis B virus; HCV, hepatitis C virus; RR, relative risk of develping intrahepatic cholangiocarcinoma; OR, adjusted odds ratio by multiple logistic regression analysis; HR, adjusted hazard ratio by Cox proportional hazard regression analysis.

* The original figures reported in each study were rounded up to the first decimal place.

† HBV infection status verified by the antiHBc+ and HBsAg−; Odds ratio for HBsAg+/antiHBc− was shown to be 2.9 (95%CI: 0.1–236.9).

We found that the incidence rate of ICC among the apparently healthy population aged 40–64 years was roughly 1 ± 0.5 per 100 000 person-years in Osaka where the HCV carrier rate is relatively high [21]. This figure is higher than the estimated incidence rate of 0.037 in the population aged 40–69 years reported in another Japanese study [22], of which the rate was underestimated because the numerator was derived from a multi-centre survey of primary liver cancer across Japan [23,24] with limited population coverage. The point-estimate incidence rate of ICC among anti-HCV positive subjects in our study (6.34 per 100 000 person years) was not very different from the estimate in the cohort study from the USA (4.0 per 100 000 person years) [10], even though our confidence interval was quite large.

Our results suggest that HBV infection is likely to be an independent risk factor for ICC in Japan. Studies in the past, in aggregate, suggested that HBV and HCV infection are potential risk factors for ICC, but their impact might be different across different countries or areas (Table 3). Several studies from the USA and Italy [9–12,14] consistently found that HCV infection was significantly associated with ICC, while the association with HBV infection was inconsistent or not assessed. On the other hand, recent hospital-based case–control studies from Korea and Shanghai, China [7,8] found that not HCV but HBV infection was significantly associated with ICC. The most recent study from Taiwan found that both HBV and HCV are significantly associated with ICC [6]. To our knowledge, our study is the first cohort study to demonstrate a significant association between HBV infection and ICC. This higher level of evidence supports prior observations of the association in East Asia. Our finding is also supported by the results of the earlier mentioned multi-centre primary liver cancer survey in Japan, which consistently showed a high prevalence of HBsAg among ICC cases (4–9%) since 1990 [24][detailed data since 1990 available in reports by the The Japan Society of Hepatology (Japanese only)].

The mechanism of carcinogenesis by HBV in intrahepatic bile ducts has not yet been elucidated, but HBV infection is an established risk factor for hepatocellular carcinoma. Because both hepatocytes and cholangiocytes differentiate from the same progenitor cells, HBV might induce carcinogenesis in both cell types through the same mechanism. The HBV gene has been detected in cholangiocarcinoma tissue in some studies [25,26], and its presence has been associated with the potential of carcinogenesis in human cholangiocytes [27]. Alternatively, hepatitis-associated ICC may arise from hepatic progenitor cells, as suggested by Lee *et al.* [6].

In our study, the association between ICC and HCV infection was not significant, even though its hazard ratio was greater than unity. We might have seen a significant association if our cohort had been even larger. In the multi-centre survey in Japan, the prevalence of anti-HCV among ICC cases (20–30%) has consistently been high since 1990 [24]. [detailed data since 1990 available in reports by The Japan Society of Hepatology (Japanese only)].

Our findings also suggest that liver inflammation as expressed by ALT level ≥40 KU may be an independent risk factor for ICC after adjustment for HCV and HBV infection status. In our previous study using the same cohort, we also found that liver inflammation is an independent risk factor for hepatocellular carcinoma [15]. The only study in the past that assessed the impact of ALT level on the development of ICC was performed in a case–control study [13], and found a significant association between ICC and ALT level of ≥40 IU, independent of HBV or HCV infection status. In addition to viral hepatitis, fatty liver disease is likely to be a major cause of liver inflammation in the subject population. Here, inflammation of hepatocytes may connote inflammation of intrahepatic bile ducts. Epidemiologic and experimental evidence shows that inflammation of the bile duct from various aetiologies induces carcinogenesis in cholangiocytes [1,4,28]. Alternatively, inflammation of hepatic progenitor cells may result in cholangiocarcinoma [6].

Liver cirrhosis has been suggested as an independent risk factor for ICC in some case–control studies where liver inflammation was not included as a covariate [7,11,12]. We were not able to assess the risk of liver cirrhosis for ICC because of lack of this information in the blood-donor database, but patients with known liver cirrhosis were excluded in our study upon screening before blood donation, minimizing the effect of a potential confounding factor. In part, the presence of liver cirrhosis among ICC cases in the case–control studies might be a proxy of present or past liver inflammation. If so, ICC might be associated with liver inflammation in cohort studies using subjects in a pre-cirrhosis state, and with liver cirrhosis in case–control studies. The only case–control study that included both liver cirrhosis and inflammation as covariates demonstrated that ICC was significantly associated with the presence of inflammation but not with liver cirrhosis [13]. Further studies on this issue are necessary.

Our study has some potential limitations. First, information on the presence of established major risk factors for ICC, namely infection with liver fluke, intrahepatic biliary stones, fibropolycystic liver disease and primary sclerosing cholangitis, was not available in the blood-donor database, and we were not able to adjust the hazard ratios for them. However, liver fluke (*Clonorchis sinensis*) infection was already rare in Osaka and surrounding areas by the 1960s [29]. Also, patients with chronic liver or autoimmune diseases were excluded from blood donors upon their claim in the screening questionnaire. Therefore, influence from known confounding factors should be minimal in our study. Second, the number of ICC cases in our study was fairly limited because of the relatively low incidence of ICC, resulting in a wide confidence interval in the estimated incidence rates or relative risks. Our findings on the risk of viral infection and inflammation, however, were consistent with observations in past studies, and it should, at least in part, warrant the validity of our findings. Third, we may have underestimated the incidence rates of ICC in our study, because the number of person-years of observation described in the Results section was not adjusted by the number of subjects who might have moved out of the province or died, before the final date of follow-up. The estimated incidence rates, after adjustment using the revised values for these parameters, would be approximately 25% higher than the figures described earlier. Lastly, coverage of the cancer registry in Osaka was not perfect, potentially reducing the estimated incidence of ICC. Nevertheless, we assume that the coverage for cancers with poor prognosis, like ICC, was sensitive enough because of the use of death certificate information. Also, the imperfect coverage of the registry should not affect the relative risk for HBV/HCV infection or liver inflammation because the registry coverage was independent of the presence of these risk factors.

In conclusion, our results suggest that HBV infection and liver inflammation are independently associated with ICC development. These findings as well as their association with liver cirrhosis and other potential risk factors need to be verified by further large cohort studies.

## Statement of interests

Author’s declaration of personal interests: None of the authors claim potential conflict of interest that pertains to this manuscript.

Declaration of funding interests: This study was funded in part by a Grant-in-Aid for Cancer Research and a Grant-in-Aid for the Third Term Comprehensive 10-Year Strategy for Cancer Control from the Japanese Ministry of Health, Labor and Welfare, and by a Grant-in-Aid for Scientific Research (B) from the Japanese Ministry of Education, Culture, Sports, Science, and Technology.

## Abbreviations:

HBsAg: Hepatitis B surface antigen
HBV: hepatitis B virus
HCV: hepatitis C virus
anti-HCV: antibody to HCV
HR: hazard ratio
HTLV-1: human T-cell lymphotropic virus type 1
ICC: intrahepatic cholangiocarcinoma
KU: Karmen Unit
OCR: Osaka Cancer Registry
